# MARCH1 Deletion Attenuates HFpEF by Promoting Adipose Beiging

**DOI:** 10.1002/cph4.70141

**Published:** 2026-04-15

**Authors:** Yunlong Zhu, Jie Fan, Liang Tang, Yuying Zhou, Kang Jiang, Dan Tan, Haobo Huang, Mingxing Wu, Jianping Zeng, Hui Yang, Shenghua Zhou

**Affiliations:** ^1^ Department of Cardiology The Second Xiangya Hospital of Central South University Changsha Hunan China; ^2^ Department of Cardiology The Central Hospital of Xiangtan(The Affiliated Hospital of Hunan University) Xiangtan Hunan China

**Keywords:** HFpEF, KLF15, MARCH1, WAT beiging

## Abstract

**Background:**

Membrane‐associated RING‐CH1 (MARCH1) is a critical membrane‐bound RING domain E3 ubiquitin ligase that primarily regulates immune cell development, immune responses, antigen presentation modulation, and metabolic functions. Its role in the pathogenesis of heart failure with preserved ejection fraction (HFpEF) remains elusive. This study aimed to investigate the function of MARCH1 in HFpEF.

**Methods:**

Multi‐hit (high fat/sugar diet together with deoxycorticosterone acetate and angiotensin II injection) HFpEF mouse model was established in C57BL/6 wild‐type mice and MARCH1 KO mice. Non‐HFpEF control mice received standard diet. Comprehensive evaluation was carried out through echocardiography, histological and biochemical studies. White adipose tissue (WAT) beiging was analyzed in isolated stromal vascular fraction cells. The relevant molecular mechanisms were determined through RNA sequencing and co‐immunoprecipitation assay.

**Results:**

Cardiometabolic dysregulation with hypertension and obesity was observed in WT HFpEF mice. Significantly reduced running distance and running time were found in HFpEF mice compared to the WT control mice (both *p* < 0.05). Histologic and echocardiographic examinations also visualized cardiac fibrosis, left ventricular hypertrophy, and diastolic dysfunction in HFpEF mice. MARCH1 expression was significantly upregulated in the WAT of HFpEF mice. MARCH1 deficiency alleviated cardiac dysfunction of HFpEF mice and induced WAT beiging following HFpEF. Mechanistically, MARCH1 contributes to HFpEF by direct binding to Krüppel‐like factor 15 (KLF15). Overexpressing KLF15 blocked the beiging effects of WAT from MARCH1 KO mice.

**Conclusion:**

Our study highlights the positive anti‐fibrotic and WAT beiging effects of MARCH1 inhibition in the setting of HFpEF. Targeting MARCH1 and KLF15 might serve as novel therapeutic options for HFpEF.

## Graphical abstract

Proposed mechanism of MARCH1 in regulating adipose tissue beiging and cardiac function in HFpEF. Schematic diagram summarizing the key findings of this study. In the HFpEF heart (top right), MARCH1 expression is upregulated in white adipose tissue (WAT), where it interacts with and likely degrades the transcription factor KLF15. This suppression of KLF15 inhibits the process of WAT beiging, characterized by reduced thermogenesis and lipid oxidation, thereby contributing to systemic metabolic dysfunction and diastolic cardiac impairment. In contrast, MARCH1 deletion (bottom left) alleviates the inhibition on KLF15, promoting the transformation of WAT into beige adipose tissue. This enhanced beiging, evidenced by increased thermogenic gene expression (e.g., UCP1), improves systemic metabolism and attenuates diastolic dysfunction. The rescued heart (bottom right) depicts the therapeutic outcome of MARCH1 inhibition, leading to improved cardiac function via the MARCH1‐KLF15‐beiging axis. MARCH1, an E3 ligase upregulated in adipose tissue of HFpEF, is identified as a key mediator of adipose‐cardiac crosstalk. Genetic deletion of MARCH1 improves cardiac function and induces beiging via interaction with KLF15, offering a novel therapeutic target.

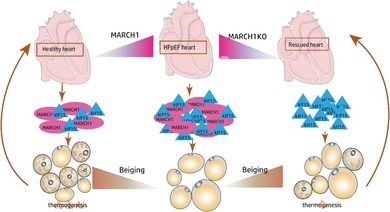

**Abbreviations**
ACC2: acetyl‐CoA carboxylase 2
ANGII: angiotensin II
AUC: area under the curve
CIDEC: cell death inducing DFFA like effector C
DOCA: deoxycorticosterone acetate
E/A: the ratio of early (E) to late (A) diastolic peak flow velocities
FAs: fatty acids
GAPDH: glyceraldehyde‐3‐phosphate dehydrogenase
HepG2: human hepatocellular carcinoma
HFHS: high‐fat high sugar diet
HFpEF: heart failure with preserved ejection fraction
IBMX: 3‐isobutyl‐1‐methylxanthine
IP: immunoprecipitation
IVS,d: end‐diastolic interventricular septal wall thickness
KLF15: Krüppel‐like factor 15
LDs: lipid droplets
LVEF: left ventricular ejection fraction
LVEF: left ventricular ejection fraction
LVPW,d: left ventricular end‐diastolic posterior wall
MARCH1: membrane‐associated RING‐CH1
MARCH1KO: membrane‐associated RING‐CH1 knockout
OGTT: oral glucose tolerance test
SVF: stromal vascular fraction
TAGs: triglycerides
UCP1: uncoupling protein 1
WAT: white adipose tissue
WT: wild type

## 1 Introduction

Heart failure (HF) represents a growing global public health challenge with increasing prevalence worldwide. Among the four recognized HF phenotypes, heart failure with preserved ejection fraction (HFpEF) has emerged as the predominant form (Redfield and Borlaug 2023). Epidemiological data demonstrate a consistent rise in HFpEF incidence over the years and poor long‐term outcomes (Owan et al. 2006; Shah et al. 2020; Zhu et al. 2025).

HFpEF is a heterogeneous syndrome primarily characterized by left ventricular diastolic dysfunction and systemic metabolic derangements (Da Dalt et al. 2023; Borlaug et al. 2025; Zhou et al. 2023). Recently, the “adipokine hypothesis” proposed by Dr. Packer offers a new perspective on this pathogenic condition. The adipokine hypothesis posits that HFpEF is an adipose‐driven disorder, originating from dysfunctional visceral adipose tissue that secretes pathogenic adipokines to the heart via endocrine or paracrine signaling (Packer 2025a, 2025b, 2026a). By placing adipose tissue to a pivotal causal role of HFpEF, this hypothesis is of significant clinical importance to handle the coexisted hypertension, diabetes, chronic kidney disease, all serve as the mainstream pathogenic driving force accelerating the development of HFpEF in the context of fat metabolism abnormalities, specifically, the imbalance of cytoprotective to proinflammatory molecular messengers secreted by visceral adipos tissue might be one of the core mechanisms of HFpEF.

Within cardiomyocytes, fatty acids (FAs) undergo compartmentalized processing: FAs may be transported into mitochondria for β‐oxidation or esterified into triglycerides (TAGs) stored within lipid droplets (LDs), and excess lipid accumulation could cause heart dysfunction (Goldberg et al. 2012). Although LDs serve as specialized organelles maintaining lipid homeostasis—a function extensively characterized in white adipose tissue (WAT) (Murphy and Vance 1999), their role in the heart is not fully understood yet. Notably, the adipokine hypothesis recognizes adipose tissue dysfunction is the root cause of HFpEF (Packer 2025a), visceral adipose tissue, a key pathogenic depot of WAT, is considered the primary culprit in this process. The continued research exploring the precise mechanisms linking adipose tissue abnormalities and the heart in the setting of HFpEF might help understand the disease feature and develop targeted medication under the highlight of the adipokine hypothesis.

The membrane‐associated RING‐CH (MARCH) protein family comprises 11 members. Structurally, all MARCH proteins contain a C4HC3‐type RING‐CH domain and multiple transmembrane (TM) domains (Zheng 2021). Membrane‐associated RING‐CH1 (MARCH1) is a critical membrane‐bound RING domain E3 ubiquitin ligase that primarily regulates immune cell development, immune responses, and antigen presentation modulation (Ohmura‐Hoshino et al. 2009). Recent studies have further unveiled MARCH1's critical role in metabolic regulation. MARCH1‐deficient mice exhibit improved insulin sensitivity and glucose tolerance, indicating its regulatory function in insulin signaling pathways (Nagarajan et al. 2016). Moreover, MARCH1‐mediated regulation of glucose homeostasis and lipid storage demonstrates sex‐dependent effects (Bhagwandin et al. 2018).

In this study, we found that MARCH1 served as a key regulating molecule in HFpEF. Our results indicated that genetically knockout of MARCH1 ameliorated diastolic dysfunction in a HFHS/DOCA/ANGII‐induced HFpEF mouse model, and the mechanism whereby MARCH1 protecting against HFpEF is mediated by promoting WAT beiging through interacting with KLF15.

## 2 Methods

### 2.1 Animal Studies

**FIGURE 1:**
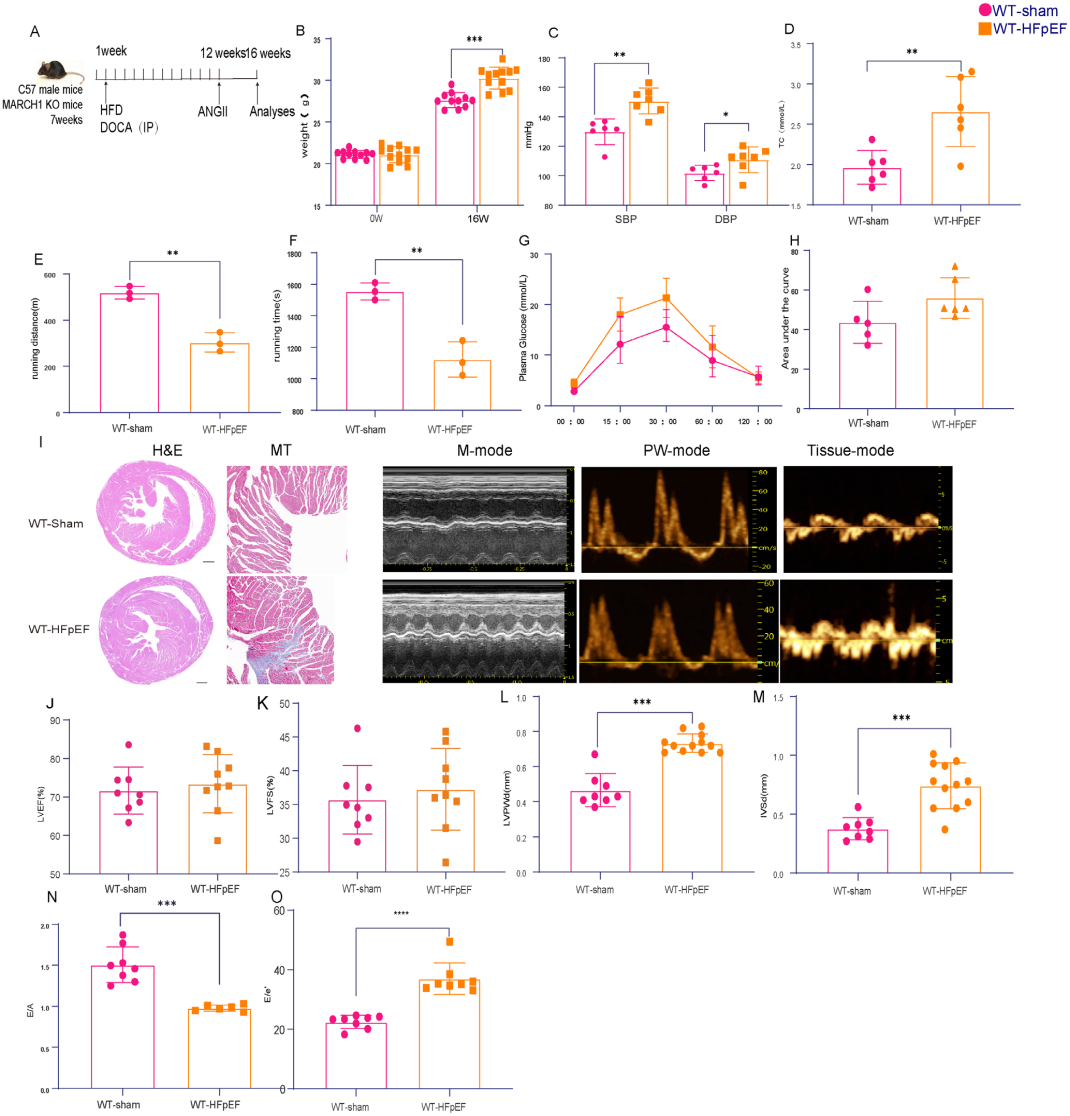
“multi‐hit” mouse model of HFpEF. (A) Schematic of the experimental setup. C57BL/6 mice were exposed to CHOW or high fat high salt (HFHS) diet and subsequently injected intraperitoneally (i.p.) with Deoxycorticosterone acetate (DOCA) at the dose of 25 mg/kg body weight or vehicle once a week for twelve weeks After that point, mice were infused with Angiotensin II (1.25 mg/kg/day) via osmotic mini pump. After four weeks, mice were subjected to functional analysis and tissue harvesting. (B) Body weight (BW) of mice of different experimental groups (*n* = 11–12 mice per group). (C) Systolic blood pressure (SBP) and diastolic blood pressure (DBP) of different experimental groups after 16 weeks of treatment (*n* = 6–7 mice per group). (D) Plasma TG levels in different experimental groups. (*n* = 6 mice per group). (E, F) Running distance and time during exercise exhaustion test (*n* = 3 mice per group). (G, H) Oral glucose tolerance test (OGTT) of different experimental groups of mice. Bar graphs depicting the area under the curve of the OGTT experiment (*n* = 5–6 mice per group). (I) Representative images of hematoxylin & eosin (H&E), Masson's Trichrome (MT) staining in transversal sections of left ventricle of mice of different experimental groups. Images are representative of three independently performed experiments with similar results. Scale bars: 500 μm (H&E),100 μm(MT). Representative left ventricular (LV) M‐mode Doppler(left) pulsed‐wave Doppler (middle) and tissue Doppler (right) tracings of WT‐sham (top panels) and WT‐HFpEF model (bottom panels) mice under basal condition (Isofluorane, Iso1.5%). (J) Percentage of left ventricular ejection fraction (LVEF). (K) Percentage of left ventricular fraction shortening (LVFS). (L) LVPWd and (M) IVSd. (N) Ratio between mitral E wave and A wave (E/A). (O) Ratio between mitral E wave and E′ wave (E/E′). Images are representative of 8–12 independent mice. Results are presented as mean ± S.E.M. *p < 0.05, **p < 0.01, ***p < 0.001.

The HFpEF model was established as previously reported (Yuan et al. 2024) with minor modifications. Adult C57BL/6 wild‐type mice and MARCH1 KO mice were fed a high‐fat high sugar diet (HFHS, 60% kcal fat, PD22072201), or a chow (CTRL; 20% kcal fat) for 12 consecutive weeks, subsequently injected intraperitoneally (i.p.) with Deoxycorticosterone acetate (DOCA) at the dose of 25 mg/kg body weight or vehicle once a week during twelve weeks. After that point, mice were anesthetized with oxygen and isoflurane (2%–3%), and a mouth mask was put over the nose and mouth of the mice to deliver this mixture throughout the pump placement, which lasted 10 min. The mice were then allowed to wake up and put back in their cage. All mice underwent surgery and an ALZET rosmotic minipump (Model 2004) was implanted in a subcutaneous pocket on the back. For sham‐treated mice, the subcutaneous pocket was closed without placement of a pump Mice were infused with angiotensin II (ANGII) (1.25 mg/kg/day) (sigma) for 4 weeks. Mice were randomized to HFHS/DOCA/ANGII‐induced HFpEF groups or sham control groups, and a total of two experimental groups were studied: (i) WT‐sham‐control diet for 12 weeks, Sham surgery; (ii) WT‐HFpEF‐HFHS/DOCA‐induced for 12 weeks with 4 weeks of ANGII‐infusion via osmotic mini‐pump. A schematic overview of the experiment is shown in Figure 1A.

### 2.2 Oral Glucose Tolerance Test

Following a previously described method (Yuan et al. 2024), a subset of mice was subjected to an oral glucose tolerance test (OGTT) in Weeks 16 (*n* = 5, 6 per group). After 12 h of fasting, mice were administered an oral bolus of glucose (2 g/kg), and repetitive tail vein blood samples were obtained at multiple time points (time from glucose bolus: 0, 15, 30, 60, 120 min). To determine glucose tolerance, we calculated the area under the curve (AUC) per treatment group.

### 2.3 Exercise Exhaustion Tests

One week prior to the experiment, animals were acclimated to treadmill exercise for three consecutive days. They ran on the treadmill at a speed of 7 m/min for 10 min daily. On the first day, mice explored the treadmill without electric stimulation. On the second and third days, mice ran on the treadmill with gradually increased speed and activated electric stimulation.

On the day of the experiment, both experimental and control groups warmed up at 5 m/min for 4 min. The treadmill speed was then increased to 14 m/min. Every 2 min thereafter, the speed was incrementally raised by 2 m/min until the animals reached exhaustion. Exhaustion was defined as the failure of a mouse to resume running within 10 s of direct contact with the electric grid or permanent residence in the fatigue zone. Total running time was recorded, and running distance was calculated. An electric stimulation of 0.4 mA was applied during the test.

### 2.4 Tail‐Cuff Blood Pressure Measurements

Systolic and diastolic blood pressure were measured noninvasively in conscious mice using the tail‐cuff method with a CODA instrument (Kent Scientific). Mice were placed in individual holders on a temperature‐controlled platform (37°C), and recordings were performed under steady‐state conditions. Prior to testing, all mice were trained to become accustomed to short‐term restraint. Blood pressure was recorded for at least two consecutive days, with readings averaged from a minimum of five measurements per session.

### 2.5 Transthoracic Echocardiography

Transthoracic echocardiography was performed using Vevo 770 System (VisualSonics, ON, Canada). Left ventricular ejection fraction and other indices of systolic function were obtained from short axis M‐mode scans at the midventricular level. Apical 4‐chamber views were used in anesthetized mice to obtain diastolic function measurements using pulsed‐wave and tissue Doppler at the level of mitral valve. Anesthesia was induced by 5% isoflurane and reduced to 1.5%. Following parameters were obtained: left ventricular ejection fraction (LVEF), the ratio of early diastolic mitral annular velocity to mitral peak velocity of late filling (E/A), end‐diastolic interventricular septal wall thickness (IVS,d), left ventricular end‐diastolic posterior wall (LVPW,d), left ventricular fractional shortening (LVFS), left ventricular ejection fraction (LVEF), peak Doppler blood inflow velocity across the mitral valve during early diastole (E), peak Doppler blood inflow velocity across the mitral valve during late diastole (A), peak tissue Doppler of myocardial relaxation velocity at the mitral valve annulus during early diastole (E′). At the end of the procedures, all mice recovered from anesthesia without difficulties.

### 2.6 Stromal Vascular Fraction (SVF) Isolation and Beige Adipocyte Induction

Wild type and MARCH1KO SVF cells were obtained from adipose tissue in the inguinal position of male C57BL/6 mice at 3–4 weeks old as previously described (Wu et al. 2024). In brief, WAT harvested was digested with collagenase I (sigma, 0.1%). SVF cells were seeded on a 6‐well‐plate and passaged when reaching 70%–80% confluence. P2 SVF cells were used for differentiation by adding induction cocktail containing IBMX (MCE), dexamethasone (Sigma), insulin (Procell), and rosiglitazone (Sigma), the medium was changed 48 h later, only containing insulin to maintain the differentiation of adipocytes, lipid droplet formation was observed on Day 7–8. To inhibit thermogenesis, differentiated cells were incubated with 100 μM propranolol for 12 h before collecting samples.

### 2.7 Co‐Immunoprecipitation Assay

Human hepatocellular carcinoma (HepG2) cells were a kind gift from Prof. Bilian Yu (The Second Xiangya Hospital of Central South University, Changsha, Hunan, China), and cells were treated with 5 mM MG132 overnight (Nagarajan et al. 2016). Cells were lysed in IP Lysis buffer (Beyotime) and lysates were used for immunoprecipitation with IgG or anti‐MARCH1 antibody and protein G‐agarose beads (MCE). To avoid interference of antibody heavy chain in immunoblot wherever possible, antibodies for immunoprecipitation and immunoblot raised in different species were chosen.

### 2.8 Adenovirus Infection

SVF isolated from 3 to 4 weeks old Wild type and MARCH1KO mice were equally seeded into 12‐well plates. Differentiated cells were infected with Ad‐Cre (Genechem). Cells were harvested 8 h after infection for Western blotting.

### 2.9 Western Blotting

Cell or tissue samples were homogenized using protein extraction buffer (Beyotime) and Protease Inhibitor. The homogenate was centrifuged at 4°C for 30 min at 14,000 *g* and the supernatant portion containing the protein fraction was isolated, avoiding the lipid fraction. Protein lysates were loaded into 12% SDS‐PAGE gels and transferred to a 0.2 μm nitrocellulose membrane and immunoblotted using UCP1 antibody (1:1000, Huabio), CIDEC antibody (1:1000, Promab), β‐actin antibody (1:5000, Proteintech), GAPDH antibody (1:300, Proteintech), and β‐Tubulin Antibody (1:1000, Proteintech).

### 2.10 Real‐Time Quantitative PCR

RNA extraction and real‐time quantitative PCR were performed as previously described. The sequences of primer pairs used in this study are shown in Table 1. Data were normalized to GAPDH and expressed as a relative ratio.

**TABLE 1:** Primer sequences used for quantitative real‐time PCR.

Genes	Sequences	Genes	Sequences
Cidec‐F	ATGGACTACGCCATGAAGTCT	Cidec‐R	CGGTGCTAACACGACAGGG
March1‐F	TTGCGCTTTGTCCACCAGT	March1‐R	GATTTCCTCCGCTGTCCGAT
Ucp1‐F	CTGGACCTTGGCCCTTTG	Ucp1‐R	GCCCCCTGCATCTGTTCT
Dio2‐F	TCCGCTACCTCAAGGACAAC	Dio2‐R	GCTGGTGGTGGTGGTGGT
Cox8b‐F	TCCGCTACCTCAAGGACAAC	Cox8b‐R	GCTGGTGGTGGTGGTGGT
Cidea‐F	CTGCCTTGCTCTTCTTCCTC	Cidea‐R	GGTAGCCCTCGTAGTCATCC

## 3 Results

### 3.1 Combination of HFHS, DOCA, and ANGII Infusion Results in Cardiometabolic Dysregulation With Hypertension and Obesity

As patients with HFpEF very often harbor the comorbidities of hypertension and metabolic dysfunction, we adopted the “multi‐hit” experimental protocol (Yuan et al. 2024) coincidence of obesity, hypertension, and dyslipidemia to establish the HFpEF model.

Male C57BL/6N wild‐type mice were divided into two treatment groups and exposed to HFHS, DOCA and ANGII infusion or standard (CHOW) diet for sixteen weeks (Figure 1A). As expected, the combination stimulation elicited body weight increases and elevated blood pressure (Figure 1B,C), elevated total cholesterol levels (Figure 1D), and significantly reduced running distance and running time compared to the wild type cohort (Figure 1E,F). However, there was no significant difference in oral glucose tolerance test (OGTT) between WT‐HFpEF and WT‐sham mice (Figure 1G,H). Besides, increased cardiac fibrosis was visualized in HFHS/DOCA/ANGII‐induced HFpEF mice (Figure 1I). The echocardiographic evaluation revealed persistent preservation of the left ventricular ejection fraction (LVEF) and left ventricular fractional shortening (LVFS) in both groups (Figure 1J,K), HFHS/DOCA/ANGII‐induced HFpEF mice presented with left ventricular hypertrophy (Figure 1L,M) and obvious diastolic dysfunction (manifested by decrease in E/A ratio and increase in the E to E′ ratio) (Figure 1N,O).

The presence of the hypertrophic response and worsened diastolic function, coupled with hypertension and obesity observed exclusively in the HFHS/DOCA/ANGII group, all those characteristics observed in HFHS/DOCA/ANGII‐induced HFpEF mice are consistent with the symptoms reported in HFpEF patients, thus, support to the notion that this is a bona fide model of HFpEF.

### 3.2 Upregulated MARCH1 Expression in the WAT of HFpEF Mice

**FIGURE 2:**
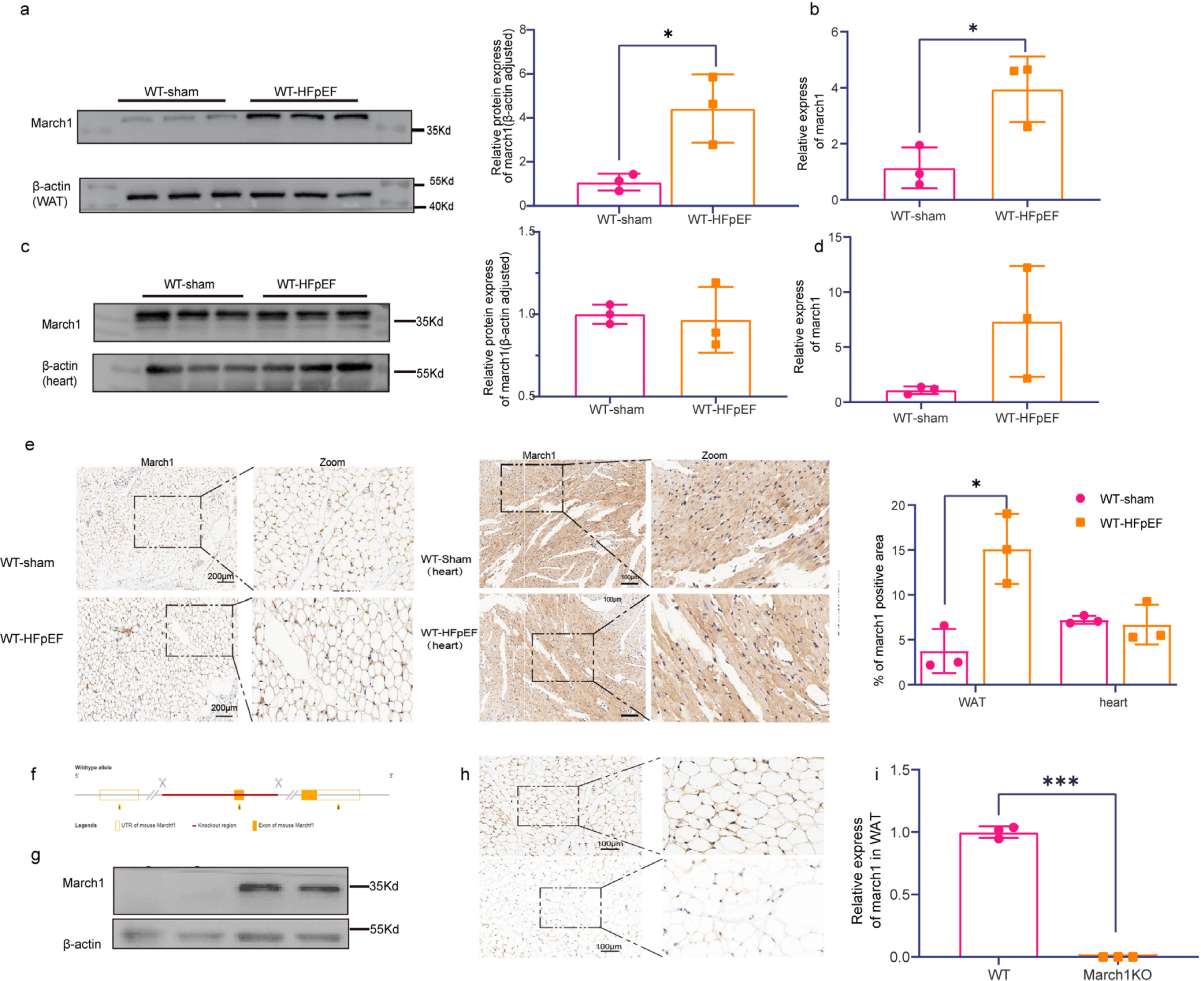
Generation and characterization of MARCH1 knockout mice. (a, b) Western blots and Real‐time PCR showing MARCH1 expression in mice WAT tissue. (c, d) Western blots and RT‐qPCR showing MARCH1 expression in mice heart tissue. (e) Representative images and quantification of MARCH1 immunohistochemistry of the WAT and cardiac sections of mice. Scale bars, 200 and 100 μm. (f) Schematic of the strategy to generate MARCH1 knockout mice. (g, h,i) Western blots and immunohistochemistry staining. Real‐time PCR showing MARCH1 expression in MARCH1 KO mice in WAT tissue. Scale bars, 100 μm. Images are representative of 2–3 independent mice.

To investigate the potential role of MARCH1 in HFpEF, we examined its expression in WAT and myocardium of HFpEF mice. Both MARCH1 protein and mRNA levels were significantly increased in freshly isolated WAT of HFpEF mice compared with WT‐sham controls (Figure 2a,b). It is noteworthy that this upregulation was specific to WAT and not observed in cardiac tissue (Figure 2c,d). Immunohistochemistry for MARCH1 also demonstrated the increase of MARCH1 expression in the WAT of HFpEF mice compared with that in the WT sham controls (Figure 2e).

Given that MARCH1 was elevated under HFpEF conditions, we next investigated whether knockdown MARCH1 could ameliorate cardiac dysfunction in HFpEF mice (Figure 2f). MARCH1 KO mice were generated and genotype was confirmed by quantitative PCR, Western blot, and immunohistochemistry staining analysis (Figure 2g–i).

### 3.3 MARCH1 Deficiency Alleviated Cardiac Dysfunction of HFpEF Mice

**FIGURE 3:**
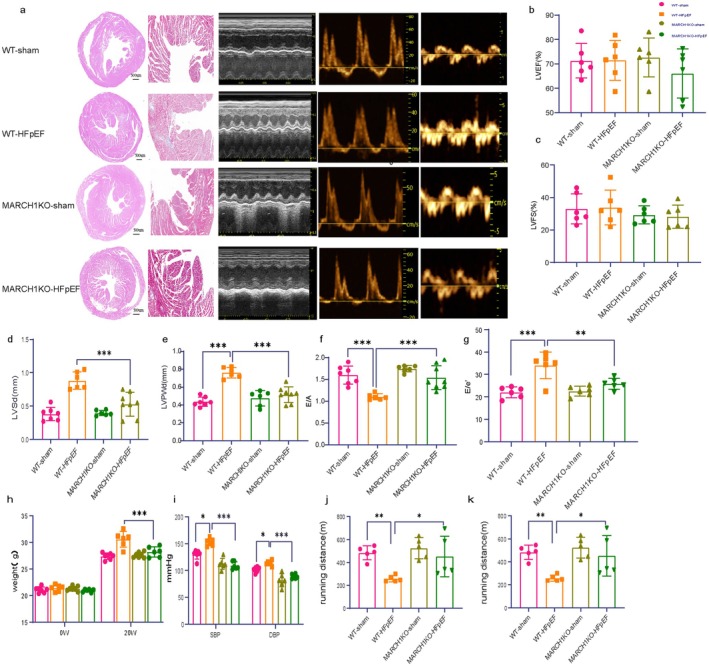
MARCH1 knockout protected against cardiac dysfunction in HFpEF mice. (a) Representative images of hematoxylin & eosin (H&E), Masson's Trichrome (MT) staining in transversal sections of left ventricle of mice of different experimental groups. Images are representative of three independently performed experiments with similar results. Scale bars: 500 μm (H&E), 100 μm (MT). Representative left ventricular (LV) M‐mode Doppler (left), pulsed‐wave Doppler (middle), and tissue Doppler (right) tracings of WT‐sham, WT‐HFpEF, MARCHIKO‐sham, MARCHIKO‐HFpEF mice under basal condition (Isofluorane, Iso 1.5%). Images are representative of six independent mice. (b) Percentage of left ventricular ejection fraction (LVEF). (c) Percentage of left ventricular fraction shortening (LVFS). (d) Left ventricular end‐diastolic posterior wall (LVPW,d) and (e) End‐diastolic interventricular septal wall thickness (IVS,d) of different experimental groups. (f) Ratio between mitral E wave and A wave (E/A). (g) Ratio between mitral E wave and E′ wave (E/E′). (h) Body weight (BW) of mice of different experimental groups (*n* = 6 mice per group). (i) Systolic blood pressure (SBP) and diastolic blood pressure (DBP) (*n* = 6 mice per group). (j, k) Running distance and time during exercise exhaustion test (*n* = 5 mice per group).

WT and MARCH1 KO mice were subjected to the HFHS/DOCA/ANGII challenges as described above, or standard (CHOW) diet as non‐HFpEF controls. MARCH1 KO HFpEF mice displayed less cardiac fibrosis and less cardiac hypertrophy as compared to WT HFpEF mice (Figure 3a,d,e). Echocardiographic analysis showed comparable cardiac systolic and diastolic function and morphology between MARCH1 KO and WT mice under physiological conditions, similar systolic function between HFpEF WT and MARCH1 KO mice (Figure 3b,c). However, markedly decreases in E/A ratio and increases in E to E′ ratio were found in WT‐HFpEF mice as compared to WT sham mice, and these diastolic dysfunction changes were alleviated in MARCH1 KO HFpEF mice (Figure 3f,g).

Body weight and systolic/diastolic blood pressure of WT HFpEF mice were increased after HFHS/DOCA/ANGII challenge, which was alleviated in MARCH1 KO HFpEF mice (Figure 3h,i). In addition, MARCH1 deficiency improved exercise intolerance (Figure 3j,k).

### 3.4 MARCH1 Deletion Induces WAT Beiging Following HFpEF

**FIGURE 4:**
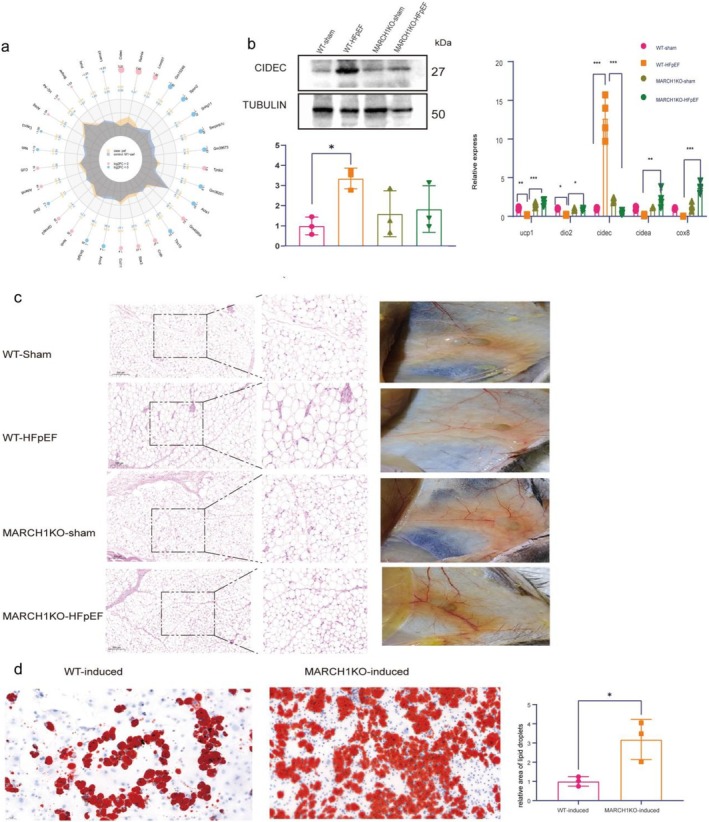
Deletion of MARCH1 ameliorates lipid metabolism in HFpEF mice. (a) Radar charts of differentially expressed genes. (b) Western blot and RT‐qPCR quantifying the expression levels. (c) Representative images of H&E stained histological sections and in situ WAT in different groups. Middle images are magnified from the indicated square in the left images. Scale bar: 200 μm. (d) Representative images of Oil Red O‐staining of lipid droplets differentiated from WT and MARCH1KO mice, *n* = 5–6.

To explore the mechanisms through which MARCH1 KO mitigates HFpEF cardiac dysfunction, RNA‐sequencing analysis was performed on WAT and cardiac tissue of WT‐HFpEF and MARCH1KO‐HFpEF to observe Differentially Expressed Genes (DEGs). In general, increased expression of cidec and retnla in WAT of MARCH1 KO HFpEF mice implicated changes in lipid metabolic regulation. Downregulated expression of dio2 (a key gene involved in thermogenesis) suggested that MARCH1 deletion might impair thermogenic capacity of WAT (Figure 4a). Western blot and RT‐qPCR confirmed above expression changes of ucp1, dio2, cidec, cidea, and cox8 (Figure 4b). In addition, the MARCH1KO‐HFpEF WAT were browner and smaller than WAT in WT‐HFpEF mice (Figure 4c). No expression changes were observed in cardiac tissues (Supporting Information).

### 3.5 MARCH1 Directly Interacted With KLF15

**FIGURE 5:**
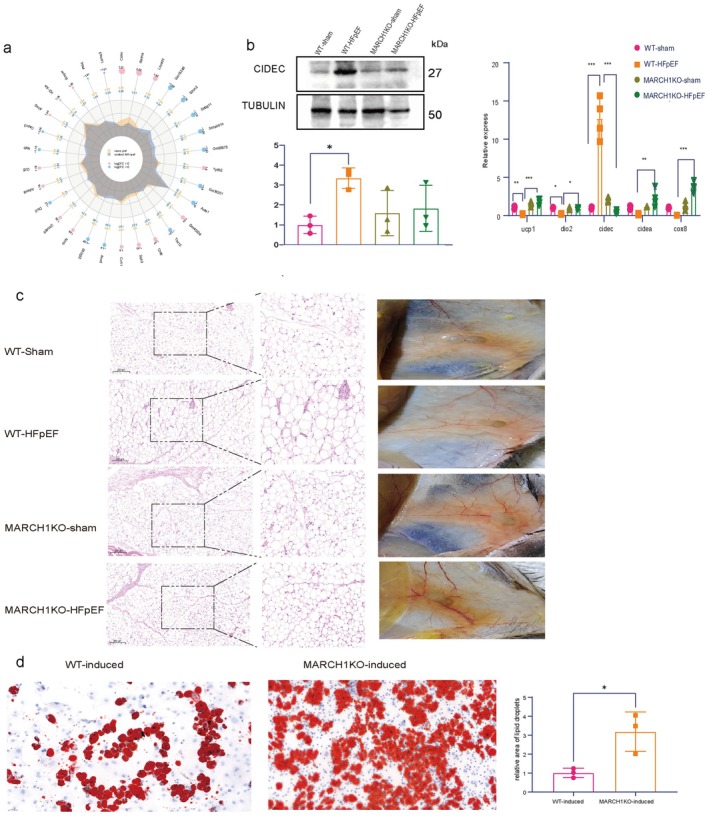
MARCH1 interacts with KLF15. (a) GSEA analysis of RNA‐seq data from MARCH1KO and MARCH1KO‐HFpEF WAT tissue. (b) Western blot quantifying KLF15 expression levels. (c) Co‐immunoprecipitation of KLF15 and endogenous MARCH1 from HepG2 cells treated with the proteasome inhibitor MG132.

To elucidate the molecular mechanism of MARCH1 knockdown in HFpEF, we performed RNA‐sequencing to screen for MARCH1‐interacting proteins, prioritizing those involved in white adipocyte differentiation. KLF15 is a transcription factor critical for adipocyte differentiation and specifically required for maintaining white adipocyte properties in subcutaneous WAT(Figure 5a). Notably, KLF15 expression was significantly reduced in MARCH1KO‐HFpEF mice compared to WT‐HFpEF mice (Figure 5b). To determine whether MARCH1 exerts its protective effects in HFpEF through direct binding to KLF15, we conducted co‐immunoprecipitation (co‐IP) assays. The interaction between endogenous MARCH1 and KLF15 was detectable in HepG2 cells, but only when MARCH1 protein stability was enhanced by the proteasome inhibitor MG132. Without proteasome inhibition, MARCH1 autoubiquitination keeps its protein levels below the limit of detection by immunoblotting (Figure 5c).

### 3.6 KLF15 Overexpression Blocked the Beiging Effects of WAT From MARCH1 KO Mice

**FIGURE 6:**
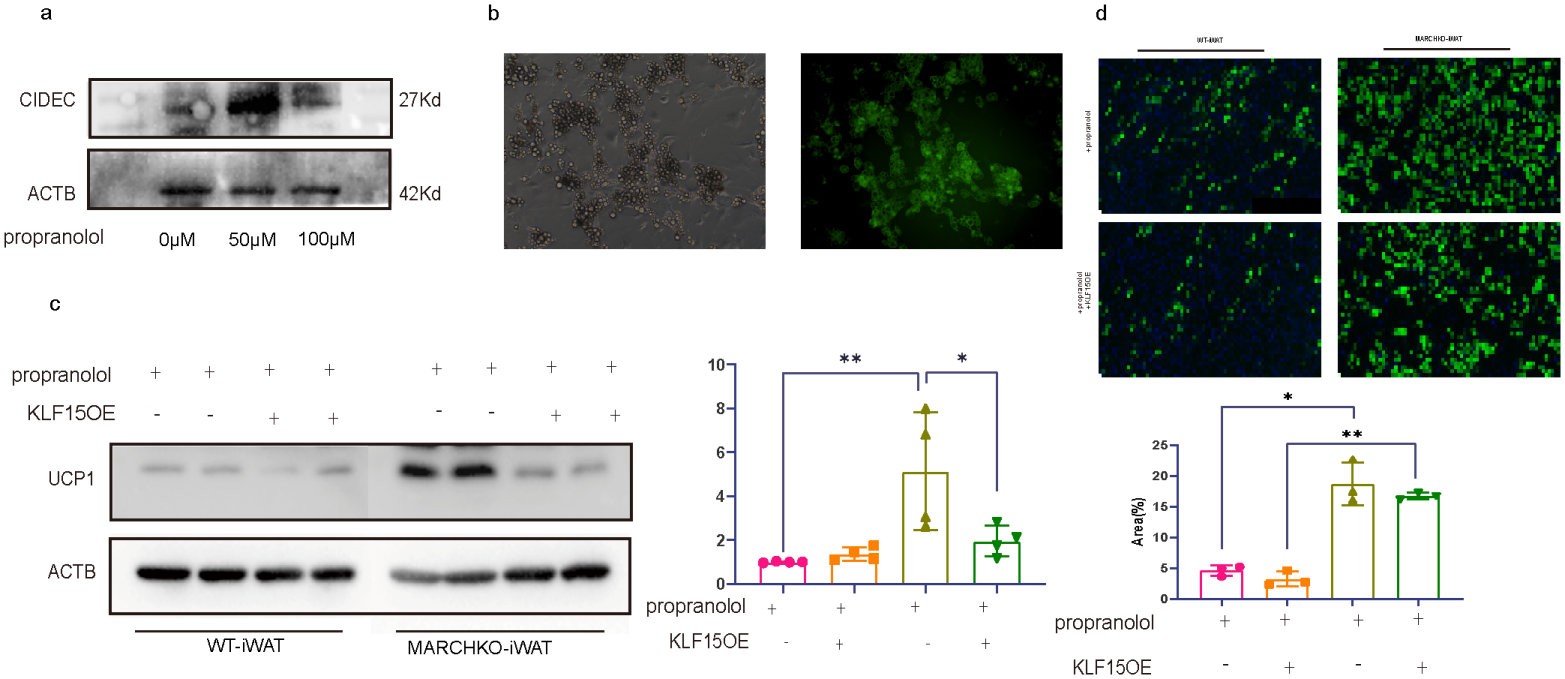
KLF15 overexpression inhibited beiging of WAT from MARCH1 KO mice. (a) Effect of varying concentrations of propranolol of cidec. (b) Fluorescence micrographs showing adenoviral transduction in cells (MOI = 400). (c) Western blot analysis of UCP1 expression following treatments with: (i) Vehicle control; (ii) Propranolol alone; (iii) Adenovirus alone (iv) Propranolol + adenovirus. (d) BODIPY staining (up) and quantitative analysis (down) of mouse beige adipocyte induction treated with propranolol or adenovirus (*n* = 3).

To further evaluate the mechanism of MARCH1 deficiency in HFpEF, we isolated WAT from MARCH1‐KO and WT mice, differentiated them in vitro, and treated them with or without propranolol before infecting with either KLF15‐overexpressing adenovirus or control adenovirus (Figure 6a,b). Results showed that KLF15‐overexpression significantly reduced ucp1 expression (Figure 6c) and reduced beiging (Figure 6d). Taken together, our results indicate that KLF15 overexpression inhibited beiging in vitro of WAT isolated from MARCH1 KO mice.

## 4 Discussion

Based on the multi‐hit HFpEF mice model (Yuan et al. 2024), present study revealed that the model reproduces key cardiometabolic features of human HFpEF, including obesity, diastolic dysfunction, exercise intolerance, cardiac hypertrophy, and myocardial fibrosis.

In this setting, we identified membrane‐associated RING‐CH1 (MARCH1), an E3 ubiquitin ligase, as a previously unrecognized regulator of systemic metabolism and cardiac function. MARCH1 expression was selectively upregulated in white adipose tissue (WAT) but not in the myocardium, and genetic deletion of MARCH1 significantly attenuated diastolic dysfunction, reduced cardiac hypertrophy and fibrosis, improved exercise tolerance, and induced WAT beiging. Mechanistically, MARCH1 directly interacts with KLF15, while KLF15 overexpression protects against beiging (central graphic). These findings suggest that MARCH1 modulates adipose‐cardiac crosstalk and metabolic remodeling in HFpEF.

### 4.1 MARCH1 and Metabolic Remodeling in HFpEF

Metabolic dysregulation, impaired substrate utilization, mitochondrial dysfunction, and lipotoxicity are key contributors to HFpEF pathogenesis (Da Dalt et al. 2023; Borlaug et al. 2025; Zhou et al. 2023; Goldberg et al. 2012; Hahn et al. 2023; Yoshii et al. 2024). Metabolic inflexibility and reduced fatty acid oxidation increase myocardial energetic stress, which drives diastolic dysfunction (Hahn et al. 2023; Yoshii et al. 2024). MARCH1 deficiency improved systemic metabolic flexibility, likely through adipose remodeling, and thereby alleviated cardiac stress. These results align with previous findings showing that metabolic interventions targeting mitochondrial pathways ameliorate HFpEF phenotypes (Yoshii et al. 2024; Zhang et al. 2025; Capone et al. 2025).

Recent data underscore that not all adipose depots contribute equally to HFpEF. Expansion of epicardial adipose tissue (EAT) is emerging as a strong predictor of disease severity and adverse outcomes, independent of BMI, via paracrine inflammation, pericardial constraint, and lipid infiltration (Dronkers et al. 2024; Whitman et al. 2025). Such observations provide important context for our findings, as promoting WAT beiging may counterbalance maladaptive signals originating from pathogenic depots like EAT.

### 4.2 Adipose Beiging as a Therapeutic Axis

A major finding of this study is that MARCH1 deletion induces WAT beiging in HFpEF. Beige adipocytes are thermogenically active and enhance lipid utilization, improving systemic metabolic flexibility. We observed increased expression of thermogenic genes (Ucp1, Cidea, Cidec) and smaller, browner adipocytes in MARCH1‐deficient mice. Notably, these changes were specific to adipose tissue, not myocardium, underscoring the importance of extra‐cardiac mechanisms.

WAT beiging is increasingly recognized as a beneficial adaptation that reduces systemic lipid burden, dampens inflammatory signaling, and enhances energy expenditure (Wu et al. 2024; Zhang et al. 2025; Capone et al. 2025). In parallel, adipokine dysregulation‐including leptin, adiponectin, and resistin‐has been implicated in mediating obesity‐related myocardial remodeling and HFpEF progression (Theodorakis et al. 2024). These pathways may converge with the MARCH1‐KLF15 axis, positioning adipose remodeling as a potential therapeutic target.

### 4.3 Mechanistic Insights: The MARCH1‐KLF15 Axis

Mechanistically, MARCH1 directly interacts with KLF15, a transcription factor critical for maintaining white adipocyte identity. KLF15 suppression is permissive for beige adipocyte differentiation. Our co‐immunoprecipitation studies confirmed the MARCH1‐KLF15 interaction, and KLF15 overexpression reversed the beiging phenotype in MARCH1‐deficient adipocytes.

Recent work further reinforces this mechanism: KLF15 was shown to be essential for maintaining white adipocyte phenotype in subcutaneous depots, and its loss drives beiging (Li and Feldman 2024). Moreover, KLF15 regulates substrate fuel selection in brown adipose tissue, promoting fatty acid oxidation (Nabatame et al. 2021). These findings provide a molecular framework through which MARCH1‐KLF15 signaling may modulate systemic energy metabolism and influence cardiac performance in HFpEF.

### 4.4 Translational Implications

Obesity and metabolic dysfunction are not only major drivers of disease severity and therapeutic response of HFpEF (Borlaug et al. 2025) but also lie at the core of the recently proposed adipokine hypothesis, which highlights that adipose tissue‐particularly visceral adipose tissue‐serves as the root of this heterogeneous syndrome (Packer 2026b). Furthermore, the STEP‐HFpEF trials demonstrated that semaglutide, a GLP‐1 receptor agonist, improved exercise capacity, natriuretic peptide levels, and diuretic requirements in obese patients with HFpEF (Kosiborod et al. 2024). SGLT2 inhibitors have similarly been shown to alter cardiac substrate metabolism, reduce inflammation, and improve microvascular function in HFpEF. The benefits of SGLT2 inhibitors (SGLT2i) in heart failure are well established, and emerging evidence suggests their molecular mechanisms extend to favorably modulating adipose tissue biology by inducing white adipose tissue (WAT) browning and improving insulin sensitivity (Morciano et al. 2024).

Collectively, these findings support the notion that adipose tissue might play a central role in the pathogenesis and therapeutic targeting of HFpEF. Using an adipose‐centric paradigm, directly reprogramming adipose phenotype might represent a promising research and therapeutic hot point of HFpEF.

Targeting MARCH1 offers a mechanistically distinct but potentially complementary strategy. Enhancing WAT beiging while mitigating maladaptive EAT signaling could improve systemic metabolic flexibility and reduce cardiac load. By acting on the MARCH1‐KLF15 axis, it may be possible to reprogram adipose phenotype to favor a metabolically healthier state.

### 4.5 Study Limitations

Our study has several limitations. First, while the multi‐hit model closely reproduces key HFpEF features, it does not encompass the full clinical heterogeneity of the syndrome, including age‐ and sex‐specific factors. Second, systemic MARCH1 deletion may have off‐target effects beyond adipose tissue; adipose depot‐specific knockout models are needed to establish causality. Third, upstream regulatory mechanisms of MARCH1 in HFpEF remain to be elucidated. Finally, translation to clinical settings will require validation in human cohorts and pharmacological targeting studies.

In conclusion, MARCH1 deletion improves diastolic dysfunction and systemic metabolic flexibility through induction of WAT beiging and modulation of KLF15 signaling. This work reveals a novel MARCH1‐KLF15‐adipose axis linking adipose biology to cardiac function in HFpEF. Together with emerging evidence on adipose depot‐specific roles and metabolic therapies, our findings highlight adipose remodeling as a promising therapeutic strategy in HFpEF.

## Author Contributions

Hui Yang and Shenghua Zhou designed the study, Yunlong Zhu and Hui Yang acquired funding, Hui Yang and Shenghua Zhou administrated the project. Yunlong Zhu and Jie Fan performed the experiments. Yunlong Zhu and Jie Fan analyzed the data. Liang Tang and Yuying Zhou drafted the manuscript. Kang Jiang, Dan Tan, Haobo Huang, and Jianping Zeng critically revised the manuscript and supervised the study. All authors contributed to the article and approved the final manuscript.

## Funding

This study was supported by the Health Commission of Hunan Province (No. R2023108), Health Commission of Hunan Province (B202303017195), Natural Science Foundation of Hunan Province (2023JJ30795). Natural Science Foundation of Hunan Province (2026JJ80123).

## Ethics Statement

The study was approved by the Ethics Committee of Second Xiangya Hospital of Central South University (No. 20231306). All procedures were performed in accordance with the National Institutes of Health Guide for the Care and Use of Laboratory Animals, and the study is reported in compliance with the ARRIVE guidelines 2.0.

## Consent

The authors have nothing to report.

## Conflicts of Interest

The authors declare no conflicts of interest.

## Supporting information


**Table S1:** cph470141‐sup‐0001‐TableS1.xls.

## Data Availability

The datasets generated and/or analyzed during the current study are not publicly available due to confidentiality agreements but are available from the corresponding author on reasonable request.
